# Child mental health in Sierra Leone: a survey and exploratory qualitative study

**DOI:** 10.1186/s13033-016-0080-8

**Published:** 2016-06-27

**Authors:** Hélène N. C. Yoder, Wietse A. Tol, Ria Reis, Joop T. V. M. de Jong

**Affiliations:** University of Amsterdam, Amsterdam, The Netherlands; Department of Mental Health, Johns Hopkins Bloomberg School of Public Health, Baltimore, MD USA; Leiden University Medical Center, Leiden, The Netherlands; Amsterdam Institute for Social Science Research, University of Amsterdam, Amsterdam, The Netherlands; The Children’s Institute, School of Child and Adolescent Health, University of Cape Town, Cape Town, South Africa; Boston University School of Medicine, Boston, USA

**Keywords:** Sierra Leone, Children, Adolescents, Mental health, Services, Healing

## Abstract

**Background:**

This study complements the growing amount of research on the psychosocial impact of war on children in Sierra Leone by examining local perceptions of child mental health, formal and informal care systems, help-seeking behaviour and stigma.

**Methods:**

The study combined: (1) a nationwide survey of mental health care providers, with (2) exploratory qualitative research among service users and providers and other stakeholders concerned with child and adolescent mental health, with a particular emphasis on local explanations and stigma.

**Results:**

Formal mental health care services are extremely limited resulting in an estimated treatment gap of over 99.8 %. Local explanations of child mental health problems in Sierra Leone are commonly spiritual or supernatural in nature, and associated with help-seeking from traditional healers or religious institutions. There is a considerable amount of stigma related to mental disorders, which affects children, their caregivers and service providers, and may lead to discrimination and abuse.

**Conclusions:**

Child and Adolescent Mental Health (CAMH) care development in Sierra Leone should cater to the long-term structural effects of war-violence and an Ebola epidemic. Priorities for development include: (1) the strengthening of legal structures and the development of relevant policies that strengthen the health system and specifically include children and adolescents, (2) a clearer local distinction between children with psychiatric, neurological, developmental or psychosocial problems and subsequent channelling into appropriate services (3) supplementary CAMH training for a range of professionals working with children across various sectors, (4) specialist training in CAMH, (5) integration of CAMH care into primary health care, education and the social welfare system, (6) further research on local explanations of child mental disorders and the effect they have on the well-being of the child, and (7) a careful consideration of the role of religious healers as care providers.

**Electronic supplementary material:**

The online version of this article (doi:10.1186/s13033-016-0080-8) contains supplementary material, which is available to authorized users.

## Background

Recent years have seen an increasing awareness of the impact of child mental health problems on the global burden of disease [1–3]. This is particularly true for low and middle income countries (LMICs), where children and adolescents constitute a high proportion of the overall population [2]. Children in low-resource settings are often disproportionally affected by risk factors such as violence, poverty, malnutrition and ill-health. Exposure to these multiple and often cumulative risks can have lasting effects on their development, mental health and psychosocial wellbeing, including a higher life-long risk of mental health problems [4–6]. Despite the vast needs in LMICs, child and adolescent mental health care is an often neglected area [1–3, 7]. In humanitarian settings, which disproportionately affect LMICs, mental health and psychosocial support programs are becoming a standard part of humanitarian interventions, but there remains a large gap between popular interventions and knowledge on effective practices [8].

In this study we examine the particular situation of child and adolescent mental health (CAMH) care in the post-conflict setting of Sierra Leone. The study was undertaken before the ebola virus disease (EVD) Outbreak of 2014/2015 and the situation on the ground will have changed in some respects. Nonetheless, we believe the findings of this study are important to take into account both for the longer term EVD outbreak response and continued attention to long-term mental health consequences of the conflict.

Situated on the west coast of Africa, Sierra Leone is bordered on the north by Guinea and on the south by Liberia. Its estimated population is approximately 6 million [9]. Sierra Leone gained its independence from Britain in 1961. From 1991 to 2002 the country experienced a brutal armed conflict, which left approximately 70,000 people killed and about half of its population displaced [10]. Human rights atrocities included the amputation of limbs, and the systematic sexual abuse of women and girls [11]. Nearly 7000 children are believed to have been recruited as child soldiers [12]. The conflict severely damaged the country’s infrastructure: health facilities were destroyed and many health professionals fled the country [13].

The 2010–2015 National Health Sector Strategic Plan of the Government of Sierra Leone paints a rather grim picture of the general maternal and child health status of the country before the EVD outbreak, including high maternal, infant and under-five mortality rates and high incidences of malaria, malnourishment and stunting of growth [14]. To tackle the many health issues, the President of Sierra Leone launched an ambitious free healthcare initiative in 2010 for pregnant women, breastfeeding mothers and all children younger than 5 years. While the programme resulted in increased healthcare use, it also revealed weak components in the health care system such as a lack of medication, diagnostic services, electricity, running water and transportation [15]. The EVD outbreak exposed the weak health systems to the outside world [16]. The epidemic resulted in a major setback in maternal and child health, due to the partial closure of health facilities, fear among the population to use health facilities, and the suspension of vaccination campaigns resulting in the emergence of old diseases such as measles [17–19].

The status of mental health care in Sierra Leone 10 years after the ending of the conflict was described by Shackman and Price [20]. They point out that at the end of the war, mental health and psychosocial support interventions focused on reconciliation, child soldier reintegration and trauma-related counselling. Many services were provided by international non-governmental organisations (NGOs), most of which left the country after completing their projects. In recent years there has been an increased local interest in the development of mental health care services in Sierra Leone. The Mental Health Coalition of Sierra Leone was initiated in 2011, bringing together national and international stakeholders in mental health [21]. The Mental Health Policy enacted in 2012 offers a framework for the development of mental health systems in the country [22]. Belfer identifies the development of CAMH policy as a key to the establishment of child and adolescent mental health care services [23]. The decision to design an addendum to the Mental Health Policy for Child & Adolescent Mental Health (with corresponding guidelines for delivery of care) in the Mental Health Strategic Plan 2014–2018 [14] can therefore be seen as a step in the right direction. References made to mental health in other documents recently released by the Government of Sierra Leone [24, 25] seem to indicate an increased awareness about mental health among law and policy makers. This may pave the way to the development of specific policy for CAMH care in Sierra Leone. At the time of our survey, the country had one retired psychiatrist, one clinical psychologist in private practice and four psychiatric nurses (one expatriate). Degree courses in psychiatry, psychology and mental health social work were unavailable [26] and there was no specialist training in CAMH. The Mental Health Strategic Plan 2014–2018 outlines strategies for the development or revision of mental health curriculum for all levels of training in health and social work, and mentions CAMH as a specific topic to be included [27]. In 2012, with help from international donors, the College of Medicine and Allied Health Sciences launched a certificate and diploma course in mental health nursing. Since the completion of our survey, 22 nurses were trained and subsequently employed in district hospitals across the country. Despite these developments, a WHO Consultation Meeting in Freetown in 2015 concluded that the country was not sufficiently prepared to deal with the psychosocial effects of the EVD outbreak and that the international response again tended to focus on short-term solutions rather than on sustainable development of a mental healthcare system [28].

A concise review of recent (January 2000–October 2015) literature on child mental health in Sierra Leone (see Additional file 1) shows that 26 out of 42 studies are exclusively on children associated with the armed forces [29–54], while another 14 included or exclusively studied the larger population of children affected by the armed conflict [55–68]. The studies include unique longitudinal research of the mental health outcomes of children associated with and/or affected by the war by Betancourt et al. [29–34, 49–51, 56, 57, 61–66, 69]. There are many valuable lessons to be learned from these studies of child mental health in Sierra Leone in the context of war, which included the linguistic and cultural adaptation of relevant assessment tools, and the development of a promising low-cost intervention to improve youth mental health and (school) functioning in war-affected youth. We identified two studies published on child mental health topics not related to the conflict [70, 71] and one prevalence study on the mental health and psychosocial needs of children with and without parental support in the eastern part of Sierra Leone [55]. Five to six years after the conflict, this study found an extremely high prevalence of mental disorders among the children (8–20 years), e.g. Major Depressive Disorder (80 %), PTSD (76.5 %) and conduct disorder (20.2 %). The symptoms experienced by these children severely impaired their social and educational capacities. Although the study has some methodological limitations, its outcomes do highlight the need for research on child and adolescent mental health in Sierra Leone.

The overall aim of this study was to identify potential barriers and opportunities for the development of CAMH care in Sierra Leone. In particular, we were interested in (a) providing an overview of current systems of child mental health care; (b) make a preliminary inventory of local explanations related to child mental health; and (c) explore how these explanations affect help-seeking patterns, and how they relate to stigma.

The WHO proMIND profile on Sierra Leone [26] identifies stigma as a major issue affecting mental health in Sierra Leone. Brief surveys done by an international NGO showed that most inhabitants of local communities believed mentally ill people to be evil, violent, lazy, stupid, unable to marry or have children, and unfit to vote [72]. In Sierra Leone society, children hold the lowest social status and their main role is to serve the household and be obedient to fathers and elders in general [73]. Therefore, behavioural and emotional deviance is likely to be less tolerated in children than in adults. This can result in an even greater stigma affecting children with mental health problems [74]. Although our study did not have stigma as its main focus, we did find it helpful to look at the many references to stigma in the light of Mukolo’s suggested framework of stigma experience in child mental disorders [75]. Mukolo’s framework describes the interrelated constructs of (1) dimensions (stereotypes, discrimination and devaluation), (2) context (self, general and public) and (3) targets (child, family/associates and service) of stigma.

## Methods

For this study, we combined a nationwide survey of mental health care providers with exploratory qualitative research among service users and providers and other stakeholders concerned with child and adolescent mental health. Data collection took place between April 2012 and May 2013.

### Sample

For the survey we included all Mental Health Care (MHC) Providers identified by and/or involved with the Mental Health Coalition of Sierra Leone in March 2012 (n = 13). An MHC Provider could be a single counsellor in private practice or an organisation with multiple staff members. For the exploratory qualitative study we used non-probabilistic sampling methods, with an emphasis on purposive sampling [76]. We used the first author’s extensive knowledge of the country and her close relationships with many key stakeholders in mental health to identify the most relevant informants in the field of CAMH. We selected informants from all four provinces in Sierra Leone. To find informants we used contact details as provided in a database of the Mental Health Coalition and/or asked local MHC Providers that were visited to assist with identification (e.g. “Can you introduce us to two local pastors or traditional healers who are known to be working with people/children with mental disorders?”). The Government-supported Christian primary and Muslim secondary schools in the Northern Province were identified by MHC Providers, while the schools in Freetown (Western Area) were identified by the first author, selecting a Government school and a Government-supported, NGO-monitored school. Purposive sampling was also used to assure gender balance. Convenience sampling was occasionally used when choices were refined based on accessibility (e.g. the possibility of being introduced through a trusted person), availability at the time of visit, or practical considerations (e.g. travel time). In an iterative process, the sample was adjusted over time: some sub-groups were reduced as less in-depth information was retrieved from them (e.g. health care providers and teachers), while others were added (e.g. parents of children with intellectual disabilities). (Head) teachers from the two government-supported schools for children with special needs were included. All informants were met at their home or place of work. All interviewed parents were living in Freetown and approached through/interviewed at the School for Special Needs (n = 4) and the Epilepsy Association of Sierra Leone (n = 1). Participants in the Group Interview were recruited voluntarily at a training session of the Mental Health Coalition in Freetown. The group included 3 MHC providers who also participated in the survey and a Traditional Healer who had facilitated visits to other healers. Table 1 gives an overview of the final qualitative research sample.

**Table 1:** Study sample qualitative research

Category	N	Male	Female	Comments
(Primary) Health care providers	10	6	4	4 Paediatricians (1 expat); 2 Doctors (1 expat); 4 Nurses
Christian healing Ministry workers	5	3	2	
Traditional healers	8	6	2	
Teachers special needs schools	2	1	1	
Teachers primary and secondary schools	4	2	2	2 Primary; 2 Secondary; 1 Teacher also trained as counsellor
Parents/caregivers	5	1	4	
Group interview	10	8	2	
Total	44	27	17	

### Tools

For the survey we used a questionnaire (see Additional file 2) which was an adapted version of tools provided in a WHO–UNHCR toolkit for the assessment of mental health and psychosocial needs and resources in humanitarian settings [77]. Data were retrieved personally by the first author (n = 11) or electronically (n = 2). Case-load information was where possible gathered through treatment records. For the qualitative research we combined semi-structured questionnaires (see Additional files 3, 4, 5, 6, 7 and 8), which were again mostly adapted versions of tools provided in the above mentioned WHO–UNHCR toolkit [77], with open interviews and a group interview. For the Group Interview we used a structured list of questions on CAMH related topics, drawn up by three of the authors (see Additional file 9). When opportunities occurred, respondents were asked about local expressions for CAMH problems in the Krio language. The interviews were conducted by the first author in Krio or English. One traditional healer had a limited understanding of Krio, so her granddaughter assisted with translation. Notes were taken during the interview in English and Krio and subsequently written out, mostly in English, and where relevant (e.g. expressions) in Krio. Outcomes of the group interview were recorded by the Interviewer (first author) on a flip chart and by a note-taker on a laptop.

### Analysis

For the survey, data were organised by question on Excel spread sheets. To make comparisons possible, some of the answers given by respondents were categorised based on agreement between authors. For the exploratory qualitative research, our analysis was thematic, using the framework method [78–80]. For each reference group, gathered and transcribed data were manually charted into a matrix (using excel spreadsheets), organised according to the questions asked. Where relevant, data were summarised in codes, based on agreement between authors. This allowed comparison and identification of information related to the two central themes local explanations and stigma, which was then charted into a separate spreadsheet and content analysis was applied. While the survey of mental health care providers was mostly aimed at gathering quantitative information, during the face-to-face meetings qualitative information related to local explanations or stigma would sometimes come up. Notes were made of the details which were later included in the analysis. To evaluate the validity of our analysis preliminary research findings were presented in a meeting with 17 key informants (including 10 members of the Mental Health Coalition of Sierra Leone) for feedback, correction and additional information (cf. Spencer et al. [81]). For this member check we used a four-point scale. Participants were asked to estimate the accuracy of the findings (see Additional file 10). Short discussions took place on different key themes. The results from this Feedback Meeting were taken into account in the final analysis and caution was exercised when data or conclusions were questioned by the group.

## Results

### CAMH in Mental Health Care Services

By March 2012, the Mental Health Coalition of Sierra Leone had identified 11 MHC Providers, functioning on 13 locations, of which seven were based in Freetown. There were no MHC Providers in the Southern Province. Additionally, two government-supported schools (one in Freetown, one in Bo) provided education for children with intellectual disabilities. Most MHC Providers made use of counsellors for whom they provided in-house training. Interviews and reviews of patient records revealed that the MHC Providers primarily served the adult population. The child and adolescent caseloads of MHC Providers over the year 2011 were low (see Table 2). The majority (84 %) of children were registered with one NGO providing group and individual counselling in the Eastern Province of Sierra Leone.

**Table 2:** Child and adolescent caseload mental health care providers

MHCP	Location	Type of Care	Child and Adolescent Caseload				
			0–12 male	0–12 female	13–17 male	13–17 female	Total
1	West	Outpatient	0	0	15	14	29
2	West	Outpatient	0	2	1	4	7
3	West	Outpatient	No information available				
4	West	Residential, day care^a^	1	0	2	0	3
5	West	Residential	No information available				
6	West	Daycare	Not yet functioning in 2011				
7	West	Outpatient	0	0	0	0	0
8	East	Outpatient^b^	121	58	52	66	297
9	East	Outpatient^b^					
10	East	Outpatient	0	0	0	0	0
11	East	Outpatient	2	1	1	1	5
12	North	Outpatient	1	2	0	1	4
13	North	Outpatient	5	3	1	1	10
		Total	130	66	72	87	355

^a^Information on day care incomplete

^b^Includes 18-year-olds

Seven MHC providers mentioned ‘lack of resources’ as a challenge, which indicates that both the sustainability and coverage of mental health care services, including services for children, remain a challenge. Two projects that were included in this survey (3 and 6 in Table 2) recently ceased their activities.

### CAMH in (primary) health care

In response to an open question on observed mental health problems, nurses and doctors most frequently mentioned mental health issues and disabilities as a result of physical illness (cerebral malaria, meningitis) or trauma (accidents resulting in traumatic brain injury). Anecdotal evidence shared by the respondents showed that they were mostly referring to severe mental and neurological disabilities. Very few cases were reported in response to presented DSM-IV categories. One paediatrician pointed out that in the absence of referral mechanisms health care workers tend to ignore mental health related issues. In general, health workers did report specific risk factors for mental health, such as poverty, abuse, changing caregivers, traffic accidents, substance abuse, and teenage pregnancy.

### CAMH in schools

Similar to health care providers, teachers reported few mental health problems in their students. A trained counsellor/teacher in a large secondary school estimated that at most about 2 % percent of the children in her school (which mostly caters for less-privileged children with relatively poor academic performance) would benefit from professional mental health care. The secondary school teachers did not report any serious problems with substance abuse in their schools, a result which was questioned by the participants in the feedback meeting. While two out of the four schools had Guidance and Counselling units, three schools reported financial constraints that prevent them from providing extra care for children with special (psychosocial) needs. In the feedback meeting participants commented that Guidance and Counselling Units are usually not very effective as they are poorly financed. According to one participant they were often supported by NGOs and ceased functioning after the NGOs departed.

### Identified CAMH problems

MHC Providers commonly did not provide diagnoses in line with psychiatric classification systems or applied terminology in alternative ways. For example, one MHC Provider diagnosed three out of their four child patients (8, 10 and 12 years old) with schizophrenia, which, based on their age, observation by the first author and provided information, seemed not in line with the DSM definition of this concept. Often MHC Providers gave a description of presenting problems rather than a formal diagnosis. In other situations we were not able to access individual patient information but were given group classifications such as “children with behavioural problems.” Table 3 gives a categorised overview of CAMH problems as identified by MHC Providers in their caseloads.

**Table 3:** CAMH problems as identified by MHC Providers in their caseloads

Category	Examples
Developmental disorders	Autism, mental retardation
Mood disorders	Depression, mania
Psychotic disorders	(Acute) psychosis, drug-induced psychosis, schizophrenia, mania
Behavioural problems & substance abuse	Behavioural problem/disorder, substance abuse (cannabis and alcohol), gang activity and membership, addiction to stealing, murder, stubbornness
Emotional Problems	Anger, low self-esteem, grief, stress, exam-related stress, rebellion, anxiety
Social Problems	Problems in social interaction
Family and child rearing issues	Family (relational) problems, rejection, “adolescence crisis” (being misunderstood by parents who do not accept their children are growing up), lack of parenting skills
Abuse	Emotional abuse, sexual harassment, rape
Issues related to sexuality	Homosexuality, masturbation, prostitution
Other	Bitterness, “unforgiveness”, financial problems, career issues, epilepsy

Most MHC providers treated children and adolescents through individual or group counselling. The two mental health units at local hospitals seemed to mostly depend on pharmaceutical treatment. Out of the fifteen children treated for mental health problems in 2011, eleven received a first-generation antipsychotic and one an anxiolytic. For the remaining three children treatment information was unavailable.

### Treatment gap

In the absence of children treated for mental health issues in the (primary) health care system, we only used the number of children registered with MHC Providers as presented in Table 2 to identify the treatment gap for children with mental health problems in 2011. We were aware of one MHC Provider who was not included in the Mental Health Coalition data base in 2012 and therefore not included in the research. Two others were not able to provide information and one was not yet functioning in 2011. Through extrapolation we estimated the total number of children who had access to mental health care as provided by MHC Providers in 2011 to be 377 children. Unicef estimated the number of children (<18 years) in Sierra Leone at 2,965,000 in 2011 [82]. Prevalence rates obtained from community studies in other resource-poor settings vary from 8 to 16 % [83]. Based on this we can expect the number of children with mental health problems in Sierra Leone in 2011 to have been 237,200 to 474,400. This reveals an estimated treatment gap of 99.8–99.9 %. This is higher than the estimated 99.5 % treatment gap for the entire population [26].

### Traditional healers

Traditional Healers mostly reported treatment of children with problems related to witchcraft or ‘demonic’ powers. Diagnoses are being made using special, God-given capacities, observation of behaviour or bodily symptoms, and divination techniques using cowry shells, mirrors, leaves, etc. Treatment may include: secret ceremonies to pull the children from the underworld (the realm of demons and witches); herbal concoctions to drink, wash or rub on faces/bodies; establishing rules/regulations that need to be respected (e.g. not taking a bath at certain hours, as there are more demons around at that time of the day); sacrifices to pacify the demons; washing with a locally made soap or “Lasmami” (written Arabic text washed from a slate) as well as advice to parents and children. During our visit with a prominent healer we were able to witness an example of a parent seeking help for her children:

*Two children (a boy aged 8 and a girl aged 11* *years) are brought in by their mother, as both are believed to have been initiated as witches. The boy’s behaviour is described as “acting like a fool” and being playful in school. In contrast, the girl is described as being “too quiet.” When questioned publicly, both children confirm the charges.*

Some healers said that sometimes it has to be accepted that a child does not improve:

“*Some are made like that by God. We don’t go against this.”* A female healer explained how in that case care should be provided: *“We give them encouragement, put them in school, wash, dress and feed them, play with them.”* Two healers who were attached to a hospital with a mental health unit said they would refer to them. They expressed particular difficulty in treating children who are born with mental disorders.

### Christian Healing Ministries

Pastors and other workers in Christian Healing Ministries told us that children and adolescents are usually brought in for a variety of reasons, e.g. witchcraft, spiritual problems, demonic attacks, seeing devils, (witch) dreams or adolescents having spiritual husbands or wives which appear to them in dreams. The observed behaviour of these children was described in various ways, e.g.: *They begin to behave differently, they are disobedient, spoil property, are stubborn. They talk in their sleep. They wet their beds. Some start stealing. They have wet dreams. They experience continuous sickness. There can be entire lifestyle changes. They become wicked. They have no fear. They fall on the ground and begin to foam.*

Problems are identified by Christian Healing Ministry workers through counselling and listening, observation of behaviour, fasting, prayer and divine revelation. Help is mostly offered in the form of counselling, prayer and fasting (for which parents and children can be encouraged or are requested to join), “deliverance” (being released from evil forces such as demons) and Bible-teaching. One church offered residential treatment, sometimes for children as young as 9 years old. Another church said to sometimes give financial assistance to parents or children. They also help with family reintegration and advocate for education for adolescents that come to them for help. One pastor told us that when younger children do not improve, he sometimes refers them to the MHC Provider present in the same town.

### Local explanations of child mental health

The Krio expression *noto ospitul sik* is commonly used for both physical and mental ailments that are believed to be beyond treatment with allopathic medication: a sickness which cannot be healed in a hospital, because the cause of the sickness is not physical but spiritual/supernatural. An analysis of all data related to aetiology showed that CAMH problems are often considered to belong to this category. Participants frequently spoke of curses, witchcraft, demon-possession, sacrifices and the breaking of taboos. Children can be afflicted by the supernatural in various ways. They can be considered victims or perpetrators or both. People can use witchcraft against them, for instance in the context of jealousy in polygamous homes. Sometimes older people transfer their witchcraft powers to children, for example grandmothers to their grandchildren. Some children are believed to enrol themselves in occultism, but is also possible that powers from the supernatural world take the initiative. A common way for this to happen is when devils appear to a child at the water side and/or attach themselves to the child through gifts (monetary or otherwise), luring them into sorcery and witchcraft. Affliction by the supernatural can also be caused by the breaking of taboos. Both a Traditional Healer and a Christian Healing Ministry worker spoke of the risk a pregnant woman runs when washing herself outside at night: the devils who are roaming around at this time will enter the woman’s navel and disturb the child. In several contexts participants mentioned the possibility that parents had made sacrifices to demons in order to obtain power or wealth. Participants in the Group Interview explained that the Krio expression for this is “Noto fo natin”: i.e., there is always a reason why something happens.

While Traditional Healers and Christian Healing Ministry workers almost exclusively identified causes in the spiritual realm, other groups of participants also gave alternative reasons for children to develop mental health problems. Primary health care providers frequently mentioned physical causes for mental health problems: brain-damage due to accidents or physical illness (malaria, meningitis), birth injuries, the effects of some prescription drugs or the use of native herbs during pregnancy, etc. On an inter-human level, participants cited stress related to the family (change of caregivers, separation or divorce of the parents, abuse, child rearing issues) and stress related to the school or community. Participants also mentioned socio-economic factors (poverty) as a possible cause for mental health problems. Two MHC Providers and participants in the Group Interview mentioned the ongoing consequences of war as a potential cause.

One situation illustrative for the co-existence of different local explanations was observed when visiting a Christian healing Ministry. A small child, physically disabled and seemingly intellectually delayed or disabled, was lying on a mattress on the floor. The first author enquired after her condition and received the following answer:

*“She was born this way. The doctors say she is mentally retarded. But counselling by the pastor revealed that the father of the child was involved in demonic business, with an agent of darkness. The serpentine spirit then came and made the mother pregnant. This child is now the manifestation. Through prayer she is now improving.”*

### Help-seeking behaviour and the role of alternative care

The limited child and adolescent caseloads we found at MHC Providers were mentioned before. While it was difficult to obtain hard data on the caseloads of traditional healers and Christian healing ministries, both groups were able to share in-depth information about the cases they dealt with (which include physical disorders) and said children were brought to them frequently (one Christian healing ministry mentioned 15–20 children per week). Parents reported to seek help from different sources. While allopathic medication was used for physical ailments, the child was taken to traditional and religious healers for treatment of mental health problems. The financial implications of this help-seeking behaviour appeared to put a significant strain on families. This is illustrated by the case of Hawanatu (not her real name), a girl who developed autistic features after a series of seizures at a young age:

*Hawanatu’s parents took her to the church, the mosque and an herbalist. At the church, the parents had to pay 150,000 leones* (approx. $35)*. People at the church would pray for Hawanatu for many hours. They also blessed water and gave it to Hawanatu to drink. At the mosque they prayed for Hawanatu and the parents didn’t have to pay. The Muslim herbalist said it was a devil and gave them medicines (leaves) and “lasmami” (*verses from the Quran written on a slate and washed off with water, which then is believed to have healing powers)*. Hawanatu’s mother explained that herbalists can be very expensive. They can charge 1,000,000 leones (approx. $225) which after bargaining they may bring down to 700,000 or 800,000 leones (approx. $160*–*180). Hawanatu’s parents spent a significant sum of money on their daughter as they visited several places.*

During the feedback meeting several participants expressed doubts about the accuracy of our findings on the role of traditional healers in child mental health care. It was felt that the research presented a picture of traditional healers which was too positive. Participants expressed concern over the abuse they believed to be going on: high financial or material demands, chaining, beating, and keeping children under inhumane conditions. It was said that some children do not survive the “treatment”.

### Stigma

We found examples of the different dimensions of stigma as suggested by Mukolo (stereotypes, discrimination and devaluation), targeting the three groups included in the model: children with mental disorders, families/associates and service providers. The examples we present here are mostly related to children with easily perceptible cognitive limitations.

*Stereotypes* could be found in the terms used to describe children with mental disorders, e.g. wicked, stubborn, or retarded. *Discrimination* often directly affects the children. A teacher told us how children with mental disorders often face difficulties when taking public transport. Families of children with mental disorders reported being forced to move frequently. On a service level, a teacher told us how she was sometimes discriminated for working at a “Special Needs School”:

*“At times, when they look at me, they can look at me as if I am crazy. Because this is a ‘mentally retarded school’.”*

Discrimination of children with mental disorders frequently results in child abuse or exploitation. Participants told us stories about children being chained or beaten. Girls with mental retardation are believed to be at increased risk of sexual violence. Their cases are not followed-up as the children cannot give evidence. Other children are subjected to child labour; they are given *“filthy jobs to do”* (group interview).

Looking at the *context* of stigma, we found that discrimination is often brought on by the general public, but also takes place on institutional level:

*“Slow learners are beaten by the teachers and parents withdraw them from school.”* (group interview).

*Devaluation* was often targeted at the child, e.g.

*“People feel they [children with mental disorders] are outcasts. They feel they are not human beings.”* (Teacher Special Needs School)

*“Children with mental health problems are considered a curse.”* (Group Interview)

*“People feel they [children with mental disorders] are not functioning properly. They cannot contribute towards the development of the community.”* (Teacher Special Needs School)

We did not find examples of self-stigmatisation, but a mother expressed how she wrestled with the public opinion about her child:

*“They say, we gave birth to a debul* (demon)*. But it is not true. She got up and she walked. If she were a debul, she’d sit down, she won’t walk. But this one, I don’t think this is a debul.”*

Stigma as an obstacle for parents to bring their child for treatment was mentioned by one informant, an MHC Provider in Freetown.

## Discussion

In this section we will discuss the results of our research with a focus on the identification of barriers and opportunities for the development of CAMH care services in Sierra Leone.

### Systems of care

Based on our interviews with health care providers, we found that virtually no mental health care services were delivered to children within the primary health care services. The general lack of case identification and management may be due to an extremely low prevalence rate, mortality due to medical neglect of children with severe mental disorders, limited geographical or financial access to health care services, or lack of diagnostic skills to recognize child and adolescent mental health problems. On a health systems level it is possible that international health development partners and the Government of Sierra Leone, in a drive to attain the millennium development goals related to child mortality, have not given commensurate attention to the mental health needs of children. This situation is not unique for Sierra Leone; it has been observed in many other parts of Africa [83, 84]. However, as more funding becomes available and considerable attention is given to the development of general child health services (e.g. the free healthcare initiative as described earlier), opportunities could be seized to include mental health aspects in the service delivery at little additional costs. With the recent opening of mental health units in district hospitals the Government of Sierra Leone, following international developments and guidelines by the WHO [85], has started to integrate mental health into primary health care. This process was accelerated during the EVD outbreak response [28]. With adequate training of the health care providers, mental health care for children and adolescents could be included.

A risk that is attached to the integration of CAMH services in primary health care is that it can lead to an overly medical approach. This can lead to overdependence on psychotropic drugs, a risk which has also been identified in high-income countries [86]. We saw this illustrated in our study when we found that the MHC providers attached to hospitals almost entirely relied on psychotropic treatment, also for very young children. This may be an argument to shy away from the health system as primary pathway into communities and look at the social welfare system instead. However, the Child Welfare Committees, which are supposed to be at the core of the child social welfare system, were found not too long ago to be functioning poorly [87]. Integration into primary health care therefore still seems the best way forward. In addition to this, recent research recommends exploring pathways into the inclusion of mental health services in the educational system [88]. Moreover, since the determinants of child mental health often also need to be addressed outside the health or education sector (e.g. poverty, gender-based violence), we believe that the development of CAMH services should not focus solely on one or two sectors, but rather have an intersectoral approach, strengthening the healthcare, social welfare, education and juvenile justice system [2, 3, 23, 89]. In every sector a major barrier to overcome will be the absence of people trained in CAMH.

### Local explanations and help-seeking behaviour

In this study we found that local explanations for mental health problems in children in Sierra Leone are commonly spiritual or supernatural in nature. Similar findings were reported in research addressing disability in Sierra Leone [90], and mental health in other Sub-Saharan African settings [91, 92].

Explanatory views held by people will determine the type of treatment they seek [93]. When people consider child mental health problems first and foremost as spiritual problems, people will seek spiritual healing, which in Sierra Leone is provided by both traditional (often Muslim) healers and Christian healing ministries. When listening to descriptions of children treated by Traditional Healers and Christian Healing Ministries, it is possible to conclude that they are dealing with children who in allopathic medicine would be diagnosed with attention deficit hyperactivity disorder, post-traumatic stress disorder, conduct disorder, epilepsy, etc. However, the case of the little girl at the Christian healing Ministry illustrates that merely providing biomedical diagnoses does not necessarily lead to acceptance of such diagnoses. The treatment of the girl (prayer) is in line with the pastor’s suggestion and is considered to be the cause of her improvement.

We must also be aware that pastors and traditional healers in Sierra Leone greatly outnumber health professionals, which makes informal care much more accessible. At the time of our interviews, the Traditional Healers Union reported a country-wide membership of over 30 thousand. It may be argued that the calculated treatment gap of 99.8–99.9 % is another example of iatrocentric reasoning by Western researchers, as it excludes the care given by traditional healers and religious institutions [86]. However, as mentioned earlier, participants in our feedback meeting felt that the role of traditional healers was portrayed as too positive in our presentation of findings. Also, it could be argued that—although more widely available—informal care is not necessarily more affordable considering the high prices that can be charged for treatment. While religious leaders and traditional healers are an important group both as recipients and transmitters of child mental health messages, their role as care providers should be critically and sensitively assessed and determined.

### Stigma

Stigma remains a major barrier in addressing (child) mental health in Sierra Leone. As we have seen, participants often mentioned supernatural causes for mental health problems. References to the supernatural may sometimes have strong negative associations that are similar to mental health stigma, for example when children are accused of being witches, or when new-borns are regarded as the ‘product’ of witchcraft. It is therefore important to include religious leaders and healers in discussions and interventions regarding child mental health stigma in Sierra Leone.

Only one MHC provider mentioned how stigma seems to withhold parents from taking their child for treatment. Generally, stigma is considered a significant barrier to seeking professional help [94]. More research is warranted into the dynamics of this phenomenon in Sierra Leone. It may also be possible that MHC providers themselves maintain stigmatising practices that keep parents from seeking treatment. The courtesy stigma affecting mental health care providers may make it less attractive for professionals to become involved in mental health care [20]. This will subsequently negatively affect the availability of care.

While some of the professionals, healers and parents we spoke with still had many questions about the mental health problems of the children they cared for, they no longer appeared afraid of them and they emphasised the need for care and acceptance. Here too seems to apply the principle that contact with people with mental disorders diminishes stigma [95]. Empowering these people to share their experience with the community may be one way to address CAMH stigma.

### Research priorities

To put CAMH higher on the agenda, further understanding of the burden of mental health and how it is understood by various populations in Sierra Leone is critical. However, as Patel has pointed out, much research on child development and psychopathology has had a dominant Western perspective, favouring psychiatric diagnostic categories (etic perspectives) over insider (emic) perspectives [2]. Most participants in this study were not aware of classifications such as autism or ADHD. Rather, most participants spoke of deviant behaviour in behavioural or spiritual terms. To what extent this behaviour could or should be interpreted in terms of “Western” psychopathology is a question which goes beyond this paper. However, it is clear that building an accessible and sustainable child and adolescent mental health system will have to take into account the diverse views on mental health problems and solutions [83]. While international classification systems may be important for comparative or funding purposes, it is essential to further understand differing patterns of symptom presentation and the role of cultural factors in vulnerability, risk and resilience [2]. The conflicting opinions on the role of traditional healers as found in this study emphasise the need for research into the (cost) effectiveness of traditional healing in CAMH (cf. IASC Guidelines on Mental Health and Psychosocial Support in Emergency Settings, action sheet 6.4 [96]).

Future research should also give attention to context-specific issues such as the long-term (trans-generational) impact of war and trauma, the effects of severe malaria, Ebola and other communicable diseases on cognitive function [97, 98] and the issue of substance abuse. This last topic was brought up by participants in the feedback meeting who believed that despite its minimal mention in this research, it is a major issue affecting the mental health of youth in Sierra Leone [99, 100]. Lastly, research should be conducted on the development of appropriate assessment tools and the design of culturally appropriate, evidence based, low-cost interventions. This research could build on the vast resources of child mental health studies in the context of war in Sierra Leone, as mentioned in the Background section of this paper.

## Limitations

The main limitation of this research is the relatively small sample size which was not always fully randomised. However, we believe this was sufficiently mitigated by the iterative process of purposive sampling and the validation provided through the feedback meeting where the initial results were analysed and criticised. Another limitation was that the (semi-)structured questionnaires and one-off meetings with participants limited the information gathering. A major advantage however was the ability of the first author to communicate in the local language and her knowledge of local culture gained through almost a decade of living and working in the country. Lastly, we acknowledge that the lack of local knowledge about mental health created a very diverse definition of child mental health conditions, varying from common psychosocial issues to severe learning disabilities or mental disorders and neurological conditions such as epilepsy. We believe however, that since clear distinctions are generally not yet made by most people at the local level, this does not reduce the relevance of the outcomes for the broad group of children struggling with neuropsychiatric and psychosocial disorders.

## Conclusion

In the aftermath of a decade of conflict and a devastating Ebola crisis, children and adolescents in Sierra Leone are at critical risk of developing mental health problems. Unfortunately, this research confirmed that children and adolescents with mental health problems in Sierra Leone have minimal access to care. This includes children with neurological disorders (such as epilepsy) or developmental disorders. Local explanations of CAMH problems in Sierra Leone are commonly spiritual or supernatural in nature, and associated with help-seeking from traditional healers or religious institutions. There are high levels of stigma related to CAMH problems. CAMH care development in Sierra Leone should cater to the long-term structural effects of war-violence and an Ebola epidemic. Priorities for development include (1) the strengthening of legal structures and the development of relevant policies that strengthen the health system and specifically include children and adolescents, (2) a clearer local distinction between children with psychiatric, neurological, developmental or psychosocial problems and subsequent channelling into appropriate services (3) supplementary CAMH training for a range of professionals working with children across various sectors, (4) specialist training in CAMH, (5) integration of CAMH care into primary health care, education and the social welfare system, (6) further research on local explanations of child mental disorders and the effect they have on the well-being of the child, and (7) a careful consideration of the role of religious healers as care providers.

## Additional files

10.1186/s13033-016-0080-8 Literature Review.
10.1186/s13033-016-0080-8 Survey Questionnaire.
10.1186/s13033-016-0080-8 Questionnaire Primary Health Care Providers.
10.1186/s13033-016-0080-8 Questionnaire Paediatricians.
10.1186/s13033-016-0080-8 Questionnaire Traditional Healers.
10.1186/s13033-016-0080-8 Questionnaire Christian Healing Ministries.
10.1186/s13033-016-0080-8 Questionnaire Parents.
10.1186/s13033-016-0080-8 Questionnaire schools.
10.1186/s13033-016-0080-8 Questions group interview.
10.1186/s13033-016-0080-8 Feedback Meeting.

## Abbreviations

CAMH: Child and Adolescent Mental Health
DSM-IV: Diagnostic and Statistical Manual of Mental Disorders, Fourth Edition
EVD: Ebola Virus Disease
LMIC: Low and Middle Income Country
MHC: Mental Health Care
NGO: Non-Governmental Organisation
UNHCR: United Nations High Commissioner for Refugees
WHO: World Health Organisation
