# Supplementary material for: Child mental health in Sierra Leone: a survey and exploratory qualitative study
Source: Int J Ment Health Syst. 2016 Jun 27;10:48. doi: 10.1186/s13033-016-0080-8 (PMC4924306; doi:10.1186/s13033-016-0080-8)
Supplement: Supplementary file 1 — 10.1186/s13033-016-0080-8 Literature Review. [file 13033_2016_80_MOESM1_ESM.pdf]

Additional File 1 – Literature Review

Methods

Keywords: Sierra Leone, Psychosocial, Psychological, Child, Adolescent, Mental Health.

Databases: PubMed, PsycInfo, Pilots, Google Scholar. Other articles in the possession of the authors or supplied by key informants.

***Inclusion criteria**:* (a) the study contains original data or is a systematic review, (b) it concerns a study population in Sierra Leone, (c) it focuses on mental health/psychosocial wellbeing and (d) it makes specific reference to children or adolescents (0-17 years).

Book Reviews, Editorials, Interviews, Conference Abstracts or News Items were excluded.

The included studies were published between January 2000 and October 2015.

Results: (see next page)

	General CAMH							
	Children Associated with the Armed Forces							
	Children Affected by the Armed Conflict							
						Both		
1	Ardizzi*, Martina; Francesca Martini3, Maria Alessandra Umilta` 1, Mariateresa Sestito1, Roberto Ravera3, Vittorio Gallese	When Early Experiences Build a Wall to Others' Emotions: An Electrophysiological Study	1				PLoS ONE 8(4): e61004. doi:10.1371/journal.pone.0061004	Journal Article
2	Behrendt, Alice	Psychosocial needs of children without parental support in a post-conflict-area:A cross section study in the district of Kailahun in Sierra Leone			1			Research Report Plan & FHI, USAID, AWARE
3	Betancourt TS, Gilman SE, Brennan RT, Zahn I, VanderWeele TJ.	Identifying Priorities for Mental Health Interventions in War-Affected Youth: A Longitudinal Study				1	Pediatrics. 2015 Aug;136(2):e344-50. doi: 10.1542/peds.2014-1521. Epub 2015 Jul 6.	Journal Article
4	Betancourt TS, McBain R, Newnham EA, Akinsulure-Smith AM, Brennan RT, Weisz JR, Hansen NB	A behavioral intervention for war-affected youth in Sierra Leone: a randomized controlled trial				1	J Am Acad Child Ad	Journal Article
5	Betancourt TS, McBain R, Newnham EA, Brennan RT.	Trajectories of internalizing problems in war-affected Sierra Leonean youth: examining conflict and postconflict factors.				1	Child Dev. 2013 Mar-Apr;84(2):455-70	Journal Article
6	Betancourt TS, McBain R, Newnham EA, Brennan RT.	Context matters: community characteristics and mental health among war-affected youth in Sierra Leone.		1			J Child Psychol Psychiatry. 2014 Mar;55(3):217-26. doi: 10.1111/jcpp.12131. Epub 2013 Sep 14	Journal Article
7	Betancourt TS, McBain RK, Newnham EA, Brennan RT.	The intergenerational impact of war: longitudinal relationships between caregiver and child mental health in postconflict Sierra Leone.		1			J Child Psychol Psychiatry. 2015 Oct;56(10):1101-7. doi: 10.1111/jcpp.12389. Epub 2015 Feb 9.	Journal Article
8	Betancourt TS, Newnham EA, McBain R, Brennan RT	Post-traumatic stress symptoms among former child soldiers in Sierra Leone: follow-up study.		1			Br J Psychiatry. 2013 Sep;203:196-202. Epub 2013 Jul 25	Journal Article
9	Betancourt TS.	A Longitudinal Study of Psychosocial Adjustment and Community Reintegration among Former Child Soldiers in Sierra Leone.		1			Int Psychiatry. 2010 Jul;7(3):60-62.	Journal Article
10	Betancourt, Theresa S	The impact of war on child development and mental health: A longitudinal study of risk and resilience among former child soldiers in Sierra Leone.		1			In: Patel, Deepali M [Ed]; Taylor, Rachel M [Ed]. Institute of Medicine; National Research Council of the National Academies. (2012). Social and economic costs of violence: Workshop summary. (pp. 88-97). xiii, 177 pp. Washington, DC, US: National Academies Press; US. [Book; Edited Book]	Book chapter/workshop summary
11	Betancourt, Theresa S.	The social ecology of resilience in war-affected youth: A longitudinal study fromSierra Leone.				1	In: Ungar, Michael [Ed]. (2012). The social ecology of resilience: A handbook of theory and practice. (pp. 347-356). xv, 463 pp. New York, NY, US: Springer Science + Business Media; US.	Book Chapter
12	Betancourt, Theresa S.; Agnew-Blais, Jessica; Gilman, Stephen E.; Williams, David R.; Ellis, B. Heidi	Past horrors, present struggles: The role of stigma in the association between war experiences and psychosocial adjustment among former child soldiers in Sierra Leone.		1			Social Science & Medicine, Vol 70(1), Jan, 2010. pp. 17-26.	Journal Article

13	Betancourt, Theresa S.; Borisova, Ivelina I.; de la Soudière, Marie; Williamson, John	Sierra leone's child soldiers: War exposures and mental health problems by gender.		1			Journal of Adolescent Health, Vol 49(1), Jul, 2011. pp. 21-28.	Journal Article
14	Betancourt, Theresa S.; Brennan, Robert T.; Rubin-Smith, Julia; Fitzmaurice, Garrett M.; Gilman, Stephen E.	Sierra Leone's former child soldiers: A longitudinal study of risk, protective factors and mental health.		1			Journal of the American Academy of Child & Adolescent Psychiatry, Vol 49(6), Jun, 2010. pp. 606-615.	Journal Article
15	Betancourt, Theresa S; McBain, Ryan K; Brennan, Robert T.	Trajectories of externalizing problems among war-affected youth in Sierra Leone: Results from a longitudinal study				1	Aggression and Violent Behavior. Vol.19(6), Nov 2014, pp. 708-714.	Journal Article
16	Betancourt, Theresa S; Newnham, Elizabeth A; Hann, Katrina; McBain, Ryan K; Akinsulure-Smith, Adeyinka M; Weisz, John; Lilienthal, Grace M; Hansen, Nathan.	Addressing the consequences of violence and adversity: The development of a group mental health intervention for war-affected youth in Sierra Leone.				1	In Book: Raynaud, Jean-Philippe [Ed]; Hodes, Matthew [Ed]; Gau, Susan Shur-Fen [Ed]. (2014). From research to practice in child and adolescent mental health. (pp. 157-177). xv, 256 pp. Lanham, MD, US: Rowman & Littlefield; US.	Book Chapter
17	Betancourt, Theresa Stichick; Borisova, Ivelina Ivanova; Williams, Timothy Philip; Brennan, Robert T.; Whitfield, Theodore H.; de la Soudiere, Marie; Williamson, John; Gilman, Stephen E.	Sierra Leone's former child soldiers: A follow-up study of psychosocial adjustment and community reintegration.		1			Child Development, Vol 81(4), Jul-Aug, 2010. pp. 1077-1095.	Journal Article
18	Borisova, Ivelina I	Child soldiers returning home from war: Family and caregiver impact on psychosocial reintegration.		1			Dissertation Abstracts International: Section B: The Sciences and Engineering, Vol 70(11-B), 2010. pp. 7200.	Dissertation
19	Borisova, Ivelina Ivanova; Betancourt, Theresa Stichick; Willett, John B	Reintegration of former child soldiers in Sierra Leone: the role of caregivers and their awareness of the violence adolescents experienced during the war		1			Journal of Aggression,<br>Maltreatment & Trauma, 22:8, 803-828	Journal Article
20	Burman, ME, McKay S.	Marginalization of girl mothers during reintegration from armed groups in Sierra Leone.		1			Int Nurs Rev. 2007 Dec;54(4):316-23.	Journal Article
21	Clifton-Everest, Ian	Meeting the mental health needs of children who have been associated with fighting forces: Some lessons from Sierra Leone.		1			In: Forced migration and mental health: Rethinking the care of refugees and displaced persons. Ingleby, David (Ed.); New York, NY, US: Springer Publishing Co, 2005. pp. 81-96.	Book Chapter
22	Denov, M, C Gervais	Negotiating (in) security: agency, resistance, and resourcefulness among girls formerly associated with Sierra Leone's Revolutionary United Front		1			Signs: Journal of Women in Culture and Society2007, vol. 32, no. 4	Journal Article
23	Denov, Myriam	Coping with the trauma of war: Former child soldiers in post-conflict Sierra Leone.		1			International Social Work, Vol 53(6), Nov, 2010. pp. 791-806.	Journal Article
24	Denov, Myriam; Doucet, Denise; Kamara, A.	Engaging war affected youth through photography: Photovoice with former child soldiers in Sierra Leone.		1			Intervention. 10(2):117-133, July 2012.	Journal Article
25	Gupta, Leila; Zimmer, Catherine	Psychosocial intervention for war-affected children in Sierra Leone.			1		British Journal of Psychiatry, Vol 192(3), Mar, 2008. pp. 212-216.	Journal Article
26	Harris DA.	Dance/movement therapy approaches to fostering resilience and recovery among African adolescent torture survivors.				1	Torture. 2007;17(2):134-55.	Journal Article
27	Harris, David Alan	Pathways to embodied empathy and reconciliation after atrocity: Former boy soldiers in a dance/movement therapy group in Sierra Leone.		1			Intervention: International Journal of Mental Health, Psychosocial Work & Counselling in Areas of Armed Conflict, Vol 5(3), Nov, 2007. pp. 203-231.	Journal Article

28	Kline, Paul M. & Mone, Erin	Coping with War: Three Strategies Employed by Adolescent Citizens of Sierra Leone				1	Child and Adolescent Social Work Journal, Vol. 20, No. 5, October 2003	Journal Article
29	McBain RK, Salhi C, Hann K, Salomon JA, Kim JJ, Betancourt TS	Costs and cost-effectiveness of a mental health intervention for war-affected young persons: decision analysis based on a randomized controlled trial				1	Health Policy Plan. 2015 Sep 7. pii: czv078.	Journal Article
30	McKay, Susan; Veale, Angela; Worthen, Miranda; Wessells, Michael	Building meaningful participation in reintegration among war-affected young mothers in Liberia, Sierra Leone and northern Uganda.		1			Intervention: International Journal of Mental Health, Psychosocial Work & Counselling in Areas of Armed Conflict, Vol 9(2), Jul, 2011. pp. 108-124.	Journal Article
31	Medeiros, Emilie	Integrating mental health into post-conflict rehabilitation: The case of Sierra Leonean and Liberian 'child soldiers.'		1			Journal of Health Psychology, Vol 12(3), May, 2007. pp. 498-504.	Journal Article
32	Murphy, William P	Child soldiers and community reconciliation in postwar Sierra Leone: African psychiatry in the twenty-first century.		1			In Book: Akyeampong, Emmanuel [Ed]; Hill, Allan G [Ed]; Kleinman, Arthur [Ed]. (2014). The culture of mental illness and psychiatric practice in Africa. (pp. 282-310). ix, 349 pp. Bloomington, IN, US: Indiana University Press; US.	Book Chapter
33	Newnham, Elizabeth A; Pearson, Rebecca M; Stein, Alan; Betancourt, Theresa Stichick	Youth mental health after civil war: the importance of daily stressors				1	The British Journal of Psychiatry(2015)206, 116–121. doi: 10.1192/bjp.bp.114.146324	Journal Article
34	Poyrazli, Senel [Ed]; Thompson, Chalmer E [Ed].	International Case Studies in Mental Health		1			(2013). International case studies in mental health. xii, 238 pp. Thousand Oaks, CA, US: Sage Publications, Inc; US.	Book
35	Shaw, Rosalind	The TRC, the NGO and the child: young people and post-conflict futures in Sierra Leone				1	Social AnthropologyAnthropologie Sociale(2014) 22, 3 306–325.	Journal Article
36	Song S, van den Brink H, de Jong J.	Who cares for former child soldiers? Mental health systems of care in sierra leone.		1			Community Ment Health J. 2013 Oct;49(5):615-24. doi: 10.1007/s10597-013-9597-3. Epub 2013 Jan 22	Journal Article
37	Stark, Lindsay	Cleansing the wounds of war: An examination of traditional healing, psychosocial health and reintegration in Sierra Leone.		1			Intervention: International Journal of Mental Health, Psychosocial Work & Counselling in Areas of Armed Conflict, Vol 4(3), Nov, 2006. Special issue: Disarmament, demobilization and reintegration of child soldiers. pp. 206-218.	Journal Article
38	Stark, Lindsay; Ager, Alastair; Wessells, Mike; Boothby, Neil	Developing culturally relevant indicators of reintegration for girls, formerly associated with armed groups in Sierra Leone using a participative ranking methodology.		1			Intervention: International Journal of Mental Health, Psychosocial Work & Counselling in Areas of Armed Conflict, Vol 7(1), Mar, 2009. pp. 4-16.	Journal Article
39	Umilta, Maria Allessandra; Wood, Rachel; Loffredo, Francesca; Ravera, Roberto; Gallese, Vittorio.	Impact of civil war on emotion recognition: The denial of sadness in Sierra Leone.				1	Frontiers in Psychology. Vol.4 Sep 2013, ArtID 523.	Journal Article
40	Walker, Ashley	Using photovoice and participatory action research to identify factors which impede and promote health among orphans in Sierra Leone.	1				Dissertation Abstracts International: Section B: The Sciences and Engineering, Vol 69(7-B), 2009. pp. 4126.	Dissertation

41	Williamson, John	The disarmament, demobilization and reintegration of child soldiers: Social and psychological transformation in Sierra Leone.		1			Intervention: International Journal of Mental Health, Psychosocial Work & Counselling in Areas of Armed Conflict, Vol 4(3), Nov, 2006. Special issue: Disarmament, demobilization and reintegration of child soldiers. pp. 185-205.	Journal Article	2006
42	Zack-Williams, Tunde B.	Child Soldiers in Sierra Leone and the Problems of Demobilisation, Rehabilitation and Reintegration into Society: Some Lessons for Social Workers in War-torn Societies.		1			Social Work Education, Vol 25(2), Mar, 2006. Special issue: Promoting Children's Well-Being. pp. 119-128.	Journal Article	2006
			2	26	2	12	42		
