# Supplementary material for: Child mental health in Sierra Leone: a survey and exploratory qualitative study
Source: Int J Ment Health Syst. 2016 Jun 27;10:48. doi: 10.1186/s13033-016-0080-8 (PMC4924306; doi:10.1186/s13033-016-0080-8)
Supplement: Supplementary file 2 — 10.1186/s13033-016-0080-8 Survey Questionnaire. [file 13033_2016_80_MOESM2_ESM.pdf]

QUESTIONNAIRE MENTAL HEALTH CARE PROVIDERS*

1. GENERAL INFORMATION	
1.1. Name of Institution	
1.2. Geographical Location	
1.3. Date & Time of Visit	
1.4 Staff Present (Names & Positions)	
1.5. Interviewer	
1.6. Length of Visit	
1.7. Activities during Visit	

2. NUMBER OF STAFF & QUALIFICATIONS	
2.1. Psychiatrists	
2.2. Medical Doctors	
2.3. Psychologists	
2.4. Psychiatric Nurses	Qualifications:
2.5. Nurses (SRN)	
2.6. Nurses (SECHN)	
2.7. Social Workers	Educational Qualifications:
2.8. Counsellors	Educational Qualifications:
2.9. Other Mental Health Staff	Educational Qualifications:

3. SERVICES PROVIDED								
			Adult Male	Adult Female	13-17 yrs Male	13-17 yrs Female	0-12 yrs Male	0-12 yrs Female
3.1. Residential Care	Number of Beds:							
3.2. Day Care	Number of Spaces:							
3.3. Individual Counselling		Yes / No	Average No. of Sessions/Month:			Average Total No. of Sessions:		
3.4. Group Counselling		Yes / No	Average No. of Sessions/Month:			Average Total No. of Sessions:		
3.5. Parental Coaching/Counselling		Yes / No	Average No. of Sessions/Month:			Average Total No. of Sessions:		
3.6. Pharmaceutical Treatment		Yes / No	List Available Medication:					
3.7. Home Visits		Yes / No	Average No. of Sessions/Month:			Average Total No. of Sessions:		
3.8. Other (describe)								
3.9. Are services free of charge?		Yes / No	If not, how much?			Private/Out of Pocket		
3.10 Is medication free of charge?		Yes / No	If not, how much?			Private/Out of Pocket		

4. PATIENTS OVERVIEW 2011 (NEWLY REGISTERED PATIENTS)						
	Adult Male	Adult Female	13-17 yrs Male	13-17 yrs Female	0-12 yrs Male	0-12 yrs Female
4.1. Inpatient						
4.2. Outpatient						
4.3. Information obtained through:<br>- 2011 Patient Records<br>- Staff Interview						

5. MENTAL HEALTH DATA / CASE INFORMATION CHILDREN /ADOLESCENTS 2011
If records are available, record all 2011 cases 0-17 years with presenting problem, diagnosis and treatment details.<br><br>If records are not available, interview staff, using the Case Information Questionnaire for Mental Health Care Providers.

6. REFERRALS	
6.1 How are children/adolescents usually referred to your institution?	O Health Professionals (Doctors, Nurses, etc.)<br>O Teachers or Other Educational Professionals<br>O Religious Leaders (Pastors, Imams)<br>O Traditional Healers<br>O NGO's/CBO's<br>O Police<br>O Friends/Relatives/People in the Community<br>O Other:
6.2 Does your institution refer children or adolescents to other institutions or professionals?<br><br>If yes, for what services?	Yes / No<br><br>O Assessment or Treatment of Health Conditions<br>O Religious Advice and Support, Prayer<br>O Traditional Healing<br>O (Special) Education<br>O Legal Assistance<br>O Financial/Livelihood Support<br>O Other:
6.3 With what institution, organisation or professional are you working most closely together?	

7. CHALLENGES IN CHILD/ADOLESCENT MENTAL HEALTH CARE
Please share some of the constraints/challenges you are experiencing in the treatment of children and adolescents in your institution.

*) Partly based on/adapted from "Assessing Mental Health and Psychosocial Needs and Resources: Toolkit for Major Humanitarian Crises." WHO: Draft Version, May 2011.
