# Supplementary material for: Child mental health in Sierra Leone: a survey and exploratory qualitative study
Source: Int J Ment Health Syst. 2016 Jun 27;10:48. doi: 10.1186/s13033-016-0080-8 (PMC4924306; doi:10.1186/s13033-016-0080-8)
Supplement: Supplementary file 4 — 10.1186/s13033-016-0080-8 Questionnaire Paediatricians. [file 13033_2016_80_MOESM4_ESM.pdf]

MENTAL HEALTH DATA / CASE INFORMATION
PEADIATRICIANS*

**1. GENERAL INFORMATION**	
**1.1. Professional qualifications**	
**1.2. Institution**	O Government Hospital/Clinic<br>O Mission Hospital/Clinic<br>O NGO Hospital/Clinic<br>O Private Practice<br>O Other, please specify: ____________________________
**1.3. Geographical Location**	

**2. MENTAL HEALTH CASE INFORMATION**				
In your experience as a paediatrician, please indicate how often on a yearly base you see children and adolescents (0 to 17 years) with any of the following disorders:				
**Type of Disorder**	**Often**	**Occasionally**	**Seldom**	**Never**
**Mental Retardation**<br>Children/Adolescents who develop slowly and do not learn easily. They often speak late and need help to perform simple tasks that most children of their age can accomplish without assistance.				
**Learning Disorders**<br>Children/Adolescents who have specific difficulties at school, e.g. find it harder than usual to read or to do maths.				
**Communication Disorder**<br>Children who have difficulty communicating, e.g. children who stutter/stammer.				
**Pervasive Developmental Disorder (including Autism, Rett's, Asperger)**<br>Children/Adolescents who "keep to themselves", who don't seem to be interested in others and often do not speak. They often get upset when things are going different from what they want or expect. They often have an obsession with specific things, e.g. anything that glitters or spins.				
**Hyperactivity**<br>Children/Adolescents who are extremely active, who cannot sit still or concentrate well, who often act impulsively (without thinking first), who have difficulty completing tasks, and as a result struggle in school.				
**Behavioural Disorders**<br>Children/Adolescents who are more than usual disobedient, disrespectful or often cause problems in the community.				
**Eating Disorders**<br>For example: children who refuse to eat or eat strange things; adolescents who eat very little or vomit/use laxatives to prevent weight gain (until they are dangerously underweight), or adolescents who eat excessively, followed by vomiting.				
**Enuresis/Encopresis**<br>Children/Adolescents who frequently wet their beds or during the day urinate/defecate on themselves (without medical reason).				
**Anxiety**<br>Children/Adolescents who are often anxious or fearful (sometimes expressed in physical complaints).				

**Separation-Anxiety Disorder**<br>Children/Adolescents who are extremely worried about being separated from, or losing their caregivers, often resulting in refusal to be separated from their caregivers.				
**School Phobia**<br>Children/Adolescents who refuse to go to school out of fear.				
**Selective Mutism**<br>Children/Adolescents who can speak but do not speak for more than one month in certain circumstances (e.g. in school, but excluding the first month in school).				
**Hyperventilation**<br>Children/Adolescents who hyperventilate (breath superficially and often, causing dizziness or other physical complaints).				
**Schizophrenia & Other Psychotic Disorders**<br>Children/Adolescents who hear or see things that are not there, or strongly believe things that are not true. They may talk to themselves, their speech may be confused or incoherent and their appearance unusual. Other people consider them "crazy". The symptoms are not caused by drugs or alcohol.				
**Mania/Hypomania**<br>Adolescents who have a distinct (>4 days) period of persistently elevated or irritated mood. They think "big" of themselves, often don't sleep much, talk more than usual, may come up with lots of ideas, are very easily distracted, may spend more money than they should, etc. The symptoms are not caused by drugs or alcohol.				
**Post-Traumatic Stress Disorder**<br>Children/Adolescents who develop severe stress or anxiety symptoms after having experienced or witnessed a traumatic event. For example: recurrent distressing memories, flashbacks, difficulty falling or staying asleep, difficulty concentrating, afraid of situations that remind them of the trauma, easily startled, etc.				
**Child Abuse / Domestic Violence**<br>Children/Adolescents who are victims of physical abuse.				
**Sexual Violence Girls**<br>Girls who are victims of rape or sexual abuse.				
**Sexual Violence Boys**<br>Boys who are victims of rape or sexual abuse.				
**Child Neglect**<br>Children/Adolescents who are not functioning well because their physical or emotional needs are neglected by their caregivers.				
**Ritual Abuse**<br>Children who have been victims of harmful rituals related to (traditional) religion, e.g. witchcraft accusations.				
**Depression**<br>Children/Adolescents who struggle with negative feelings (hopelessness, helplessness, sadness, low self-esteem) to the extent that there is marked impairment in daily functioning. Often they complain of insomnia, low energy and difficulty making decisions. Some adolescents are more irritable than usual, some start using substances.				
**Bereavement**<br>Children/Adolescents who struggle more than usual with the loss of parents, siblings or other close relatives.				
**Unexplained Somatic Complaints**<br>Children/Adolescents who have physical complaints that cannot be medically explained (e.g. stomach pains, headaches, extreme tiredness, partial paralysis).				
**Tic Disorder**<br>Children/Adolescents who frequently display tics (sudden, rapid, repeated motor movements or vocal sounds).				
**Obsessive-Compulsive Disorder**<br>Children/Adolescents who display repetitive behaviour (e.g. hand washing, ordering, checking) or mental acts (e.g. counting, repeating words silently).				

**Substance Abuse**<br>Children/Adolescents who are dependent on drugs (including prescription medication such as diazepam) or alcohol.				
**Substance-Induced Disorders**<br>Children/Adolescents who are experiencing mental disorders (e.g. psychosis, mania) as a result of substance abuse.				
**Other:**<br>Please list any other mental health concerns you have detected in children and their frequency.				
**1.**				
**2.**				
**3.**				
**4.**				
**5.**				

*) Partly based on/adapted from "Assessing Mental Health and Psychosocial Needs and Resources: Toolkit for Major Humanitarian Crises." WHO: Draft Version, May 2011.
