# Supplementary material for: Child mental health in Sierra Leone: a survey and exploratory qualitative study
Source: Int J Ment Health Syst. 2016 Jun 27;10:48. doi: 10.1186/s13033-016-0080-8 (PMC4924306; doi:10.1186/s13033-016-0080-8)
Supplement: Supplementary file 5 — 10.1186/s13033-016-0080-8 Questionnaire Traditional Healers. [file 13033_2016_80_MOESM5_ESM.pdf]

INTERVIEW GUIDE TRADITIONAL HEALERS

1. GENERAL INFORMATION	
1.1 Name Traditional Healer	
1.2 What is your official title?	
1.3 How many years have you been practicing traditional healing?	
1.4 How did you learn to practice traditional healing?	
1.5 Geographical Location	
1.6 Date & Time of Visit	
1.7 Interviewer	

2. FREQUENCY OF CHILD/ADOLESCENT CONSULTATIONS

2.1 Children (0-11 years)

In your healing practice, how frequent are children (0-11 years) brought in for help?

O Never
O About one child per week
O More than one child per week (approximate number of cases per week: ____)
O About one child per month
O More than one child per month (approximate number of cases per month: ____)

2.2 Adolescents (12-17 years)

In your healing practice, how frequent are adolescents (12-17 years) brought in for help?

O Never
O About one adolescent per week
O More than one adolescent per week (approximate number of cases per week: ____)
O About one adolescent per month
O More than one adolescent per month (approximate number of cases per month: ____)

3. SPECIFIC CASE INFORMATION CHILDREN	
Let's talk about the younger children (0-11 years) first:	
For what kind of problems may children be brought to you?	
How do you find out what their problems are?	
What are common causes for the problems they have?	
What help do you usually offer them?<br>Probe for procedures and use of artefacts.	

What is the role of the child in the healing process?	
What is the role of the parents/guardians in the healing process?	
What payment is usually required for a consultation?	
**NB: If an interesting, specific disorder is mentioned, go to … for additional questions.**	

**4. SPECIFIC CASE INFORMATION ADOLESCENTS**	
Now let's talk about the older children (12-17 years):	
For what kind of problems may these older children be brought to you?	
How do you find out what their problems are?	
What are common causes for the problems they have?	
What help do you usually offer them?<br>Probe for procedures and use of artefacts.	
In the case of older children, what is the role of the child in the healing process?	
What is the role of the parents/guardians in the healing process?	
What payment is usually required for a consultation?	
**NB: If an interesting, specific disorder is mentioned, go to 7 for additional questions.**	

**5. REFERRALS**	
There might be times when you decide to advise the child/adolescent and its caregivers to (also) go somewhere else for help. Can you tell me where you may send them?	O Health Services (e.g. doctors, hospitals, clinics, pharmacies)<br>O School / Teachers<br>O Social Services (e.g. Ministry of Social Welfare)<br>O NGO's or CBO's working with children & adolescents<br>O Churches or Christian Healing Ministries<br>O Other, please specify ________________________________

6. IF THE CHILD DOESN'T GET BETTER	
Sometimes children/adolescents do not get well despite the help that is offered. What do you think could be reasons for this?	
What do you usually do in this situation?	

7. PROBING QUESTIONS FOR SPECIFIC PROBLEMS

**Problem 1:**	
How do you recognise this problem?	
What are the causes for this problem?	
What kind of help do you offer for this problem?	
Is the child/adolescent usually healed? Immediately or over time?	

**Problem 2:**	
How do you recognise this problem?	
What are the causes for this problem?	
What kind of help do you offer for this problem?	
Is the child/adolescent usually healed? Immediately or over time?	

**Problem 3:**	
How do you recognise this problem?	
What are the causes for this problem?	
What kind of help do you offer for this problem?	
Is the child/adolescent usually healed? Immediately or over time?	

8. PROBING QUESTIONS FOR DISSOCIATION (IF APPROPRIATE)
Have you seen children/adolescents whose behaviour or voice suddenly changes as if they become another person (or an animal) and after a while they change back into their usual selves again?
What, in your opinion, causes this problem?
What help do you offer in such cases?

*) Partly based on/adapted from "Assessing Mental Health and Psychosocial Needs and Resources: Toolkit for Major Humanitarian Crises." WHO: Draft Version, May 2011.
