# Supplementary material for: Child mental health in Sierra Leone: a survey and exploratory qualitative study
Source: Int J Ment Health Syst. 2016 Jun 27;10:48. doi: 10.1186/s13033-016-0080-8 (PMC4924306; doi:10.1186/s13033-016-0080-8)
Supplement: Supplementary file 7 — 10.1186/s13033-016-0080-8 Questionnaire Parents. [file 13033_2016_80_MOESM7_ESM.pdf]

Interview Parents

**Details Child**	
Sex:	
Age:	
Any Physical Disability:	
Probable Diagnosis according to Researcher:	

- How old was your child when you first suspected your child had some sort of problem?
- What did you do when you noticed this?
- Did you seek help? Where/from whom?
- What did they say about the problem of your child? What help did they give?
- Did you have to pay? How much?
- Has it helped you or your child? In what way?
- How do people around you (family/community) treat your child?
- What do they say about him/her? What words do they use to describe him/her?
- What do they say about *you*? Do they help you in any way?
- Is your child going to school? If not, why not?
- Do you think the government should do something for children like your child? If yes, what?
- What are your thoughts about the future of your child?
