# Supplementary material for: Child mental health in Sierra Leone: a survey and exploratory qualitative study
Source: Int J Ment Health Syst. 2016 Jun 27;10:48. doi: 10.1186/s13033-016-0080-8 (PMC4924306; doi:10.1186/s13033-016-0080-8)
Supplement: Supplementary file 8 — 10.1186/s13033-016-0080-8 Questionnaire schools. [file 13033_2016_80_MOESM8_ESM.pdf]

EDUCATIONAL INSTITUTIONS

**1. GENERAL INFORMATION**	
**1.1. Professional qualifications and Position of Person Interviewed**	
**1.2. School**	O Government Primary School<br>O Government Secondary School<br>O Private Primary School<br>O Private Secondary School<br>O Other, please specify:
**1.3 Number of Students**	
**1.4. Geographical Location**	

**2. MENTAL HEALTH CASE INFORMATION – GENERAL**
In your experience as an educational professional working with children (0-17 years), do you see children/adolescents who have mental / psychological / emotional problems for which they need help? Yes / No
If yes, please give examples:

**3. CASE LOAD**
In your school of … students, how many children do you think would benefit from professional mental health care, if this were available?

4. HELP OFFERED

What help does your school offer to children with mental/psychological/emotional problems?

5. REFERRALS

Do you refer children with mental/psychological/emotional problems, and if yes, where?

O Medical Institutions
O Mental Health Care Providers
O Church
O Mosque
O Traditional Healer
O Police / FSU
O CBO/NGO's
O Other:

6. LOCAL IDIOMS:

Please list words/phrases people use to describe children with any form of mental / psychological / emotional distress.
