# Supplementary material for: Child mental health in Sierra Leone: a survey and exploratory qualitative study
Source: Int J Ment Health Syst. 2016 Jun 27;10:48. doi: 10.1186/s13033-016-0080-8 (PMC4924306; doi:10.1186/s13033-016-0080-8)
Supplement: Supplementary file 9 — 10.1186/s13033-016-0080-8 Questions group interview. [file 13033_2016_80_MOESM9_ESM.pdf]

Group In-Depth Interview

Participants: Selection of Mental Health Coalition Members

First: define child as a person younger than 18 years of age. Or: child 0-11 years; adolescent 12-17 years.

Questions:

- Can children have mental health problems? If yes, please list some of these problems.
- Do children with mental health problems always look different from other children?
- How do people in the community see children with mental health problems?
- How do they feel about them?
- What words do they use to describe them? (try to get an understanding of the following terms: Mad/Crazy; He/she is a witch; He/she has witch; He/she is a demon; He/she has a demon)
- How do people in the community see the *parents* of children with mental health problems?
- What do people see as the *causes* of mental health problems in children?
- Where do you think parents will go for help?
- Should the government be doing something for these children and if yes, what?
- How best do you think we can develop mental health care services for children/adolescents in this country?
