# Supplementary material for: Child mental health in Sierra Leone: a survey and exploratory qualitative study
Source: Int J Ment Health Syst. 2016 Jun 27;10:48. doi: 10.1186/s13033-016-0080-8 (PMC4924306; doi:10.1186/s13033-016-0080-8)
Supplement: Supplementary file 10 — 10.1186/s13033-016-0080-8 Feedback Meeting. [file 13033_2016_80_MOESM10_ESM.pdf]

Additional File 10

CHILD & ADOLESCENT MENTAL HEALTH IN SIERRA LEONE

RESEARCH FEEDBACK MEETING – MAY 22, 2013

Results:

		Inaccurate	Mostly Inaccurate	Mostly Accurate	Accurate	No Answer	Don't Know
Question 1	Situation Analysis CAMH Care			5	10	2	
Question 2	CAMH in (P)HC			5	11	1	
Question 3	CAMH in Schools			5	12		
Question 4	CAMH & Traditional Healers	1	3	8	4		1
Question 5	CAMH & Christian Healing		1	9	7		
Question 6	Parents/Caregivers	1		3	11		2
Question 7	Help-Seeking Behaviour		1	4	12		
Question 8	Cost of Services		1	9	7		
Question 9	Aetiology			2	14		1
Question 10	Stigma			2	15		
		Fully Disagree	Mostly Disagree	Mostly Agree	Fully Agree	No Answer	Don't Know
Question 11	Development CAMH Care			1	16		

Questions:

1. What do you think? How accurately do these findings reflect the current situation of child/adolescent mental health care In Sierra Leone?

2. What do you think? How accurately do these findings reflect the current situation of child/adolescent mental health care in (primary) health care in Sierra Leone?

3. How accurately do these findings reflect the current situation of child/adolescent mental health care in primary & secondary schools in Sierra Leone?

4. What do you think? How accurately do these findings reflect the current contribution of traditional healers to the treatment of children/adolescents with mental disorders in Sierra Leone?

5. What do you think? How accurately do these findings reflect the current contribution of Christian healing ministries to the treatment of children/adolescents with mental disorders in Sierra Leone?

6. What do you think? How accurately do the sayings of these parents reflect the situation of parents of children/adolescents with mental disorders in Sierra Leone?

7. What do you think? How accurately does the help-seeking behaviour of these parents reflect the help-seeking behaviour of parents of children/adolescents with mental disorders in Sierra Leone?

8. What do you think? How accurately does the information on cost of services reflect the situation in Sierra Leone?

9. What do you think? How accurately does the information on etiology reflect the situation in Sierra Leone?

10. What do you think? How accurately does the information on stigma reflect the situation in Sierra Leone?

11. What do you think? To what extent do you agree with the conclusions/recommendations for the development of child/adolescent mental health care in Sierra Leone?
